# Thermal invisibility based on scattering cancellation and mantle cloaking

**DOI:** 10.1038/srep09876

**Published:** 2015-04-30

**Authors:** M. Farhat, P.-Y. Chen, H. Bagci, C. Amra, S. Guenneau, A. Alù

**Affiliations:** 1 Division of Computer, Electrical, and Mathematical Sciences and Engineering, King Abdullah University of Science and Technology (KAUST), Thuwal 23955-6900, Saudi Arabia; 2 Department of Electrical and Computer Engineering, Wayne State University, Detroit, Michigan 48202, U.S.A; 3 Aix-Marseille Université, CNRS, Centrale Marseille, Institut Fresnel, Campus universitaire de Saint-Jérôme, 13013 Marseille, France; 4 Department of Electrical and Computer Engineering, The University of Texas at Austin, Austin, TX, 78712, U.S.A

## Abstract

We theoretically and numerically analyze thermal invisibility based on the concept of scattering cancellation and mantle cloaking. We show that a small object can be made completely invisible to heat diffusion waves, by tailoring the heat conductivity of the spherical shell enclosing the object. This means that the thermal scattering from the object is suppressed, and the heat flow outside the object and the cloak made of these spherical shells behaves as if the object is not present. Thermal invisibility may open new vistas in hiding hot spots in infrared thermography, military furtivity, and electronics heating reduction.

The realization of electromagnetic invisibility cloaks^1^,^2^,^3^,^4^ is undoubtedly one of the most exciting and challenging applications of metamaterials^5^. In the previous decades, thanks to the astonishing development of micro- and nano-fabrication and 3D printing, this goal has got closer to reality. In 2005, Alù and Engheta proposed a transparency device that relies on the so-called scattering cancellation technique (SCT)^6^. This mechanism consists of using a low or negative electric permittivity cover to cancel the different scattering multipoles of the object to hide. This class of cloaking devices has been shown to be quite robust to changes in the geometry of objects and the frequency of operation^7^,^8^,^9^. Moreover, a recent experimental study has shown that these cloak designs can actually be realized at microwave frequencies^10^. Applications in furtivity, non-invasive sensing, and probing can be envisaged^11^,^12^, opening new directions in medicine, defense, and telecommunications. Recent findings also suggest that objects can be made invisible using the mantle cloaking technology, where a metasurface can produce similar effects with a simpler and thinner geometry. This is achieved by tailoring the surface current on the metasurface and consequently the phase of re-radiated fields^13^,^14^,^15^,^16^. It should also be mentioned here that other cloaking techniques have been put forward in the recent years based on various concepts such as conformal mapping^1^, transformation optics^2^,^3^,^17^, homogenization of multistructures^18^,^19^, active plasmonic cloaks^20^, anomalous localized resonances^21^, and waveguide theory^22^.

The concept of invisibility has been extended to other realms of physics. Cloaks capable of hiding objects from acoustic waves^23^,^24^,^25^, surface water waves^26^, flexural bending waves^27^, seismic waves^28^,^29^, quantum matter waves^30^,^31^ and even diffusive light propagation^32^,^33^ have been developed. And more recently, after the seminal work of Guenneau *et al.*^34^, invisibility cloaks for heat waves has become another exciting venue for cloaking applications^35^,^36^,^37^. Thermal cloak designs inspired by transformation optics^2^ have been subsequently proposed^38^,^39^,^40^ to control the flow of heat in metamaterial structures. Their experimental validation followed shortly^41^,^42^,^43^,^44^. Thermal cloaking may find interesting applications in modern electronics. It can be used to reduce the heat diffused from computers or to protect a specific nano-electronic component by re-directing the flow of heat. This technique can also be used for isolation in buildings to reduce the consumption of energy required in heating or cooling.

In this paper, we propose to use the concept of scattering cancellation to generate the invisibility effect for heat diffusion waves. The peculiarity of our cloak is that, unlike earlier designs, we consider both static and time-harmonic dependence (note that time-harmonic heat sources can be generated using pulsating lasers^45^). This scenario requires cancellation of two scattering orders for small objects, i.e. the monopole and dipole ones, corresponding to the specific heat capacity and the heat conductivity, respectively. Numerical simulations confirm that a scattering reduction of over 40 dB can be obtained for optimized cloak parameters. Additionally, it is shown that the proposed cloak suppresses both the near and far heat fields. We also demonstrate that coating an object with an ultra-thin layer or thermal metasurface is a viable way for scattering reduction (mantle cloaking).

## Results

### Heat diffusion waves and their dispersion relation

**Figure 1:**
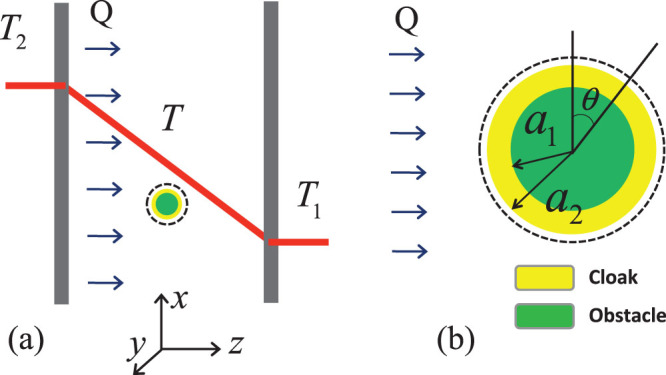
Thermal scattering problem. (a) Cross-sectional view of the heat transfer scenario, with the object to cloak in the middle. (b) Cross-sectional view of the cloaked object.

Using the first principle of thermodynamics in a closed system^46^, one can show that in the absence of radiation and convection, the temperature of a physical system obeys the Fourier relation 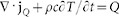. Here, 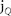, 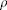 and 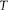 represent the density of heat flux (heat flow per unit surface per unit time), the density of the fluid, and the temperature field, respectively. 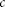 is the specific heat capacity and 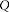 denotes the heat energy generated per unit volume per unit time (Fig. 1). Using the Fourier law, i.e. the linear and instantaneous relation 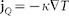, where 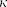 is the heat conductivity of the medium, one can derive,
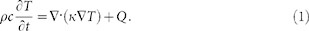
For a constant conductivity and in the absence of heat sources, Eq. (1) simplifies to 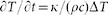. To solve this equation, one can assume that 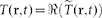, with 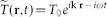, where 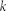 is the wave number of the *pseudo* diffusion plane wave and 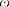 its angular frequency. This ansatz is valid, only because Eq. (1) is a linear equation, meaning that 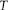 is a solution, if and only if, 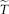 is a solution. The dispersion relation of heat diffusion waves is thus 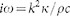. If one assumes that 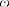 is real, then 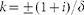, with 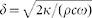. The general solutions are thus attenuated diffusing plane waves. Now, under the assumption of time-harmonic dependence 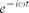, generated for instance by a pulsating laser, and constant conductivity, Eq. (1) simplifies to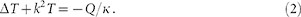 For the structure in Fig. 1, Eq. (1) is supplied with two boundary conditions that should be satisfied at the surface of both spherical object and the cloak. Across the boundaries 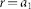 and 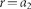, we have the continuity of the temperature and the density of heat flux, i.e. 
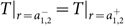
, and 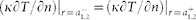, where the signs + and − refer respectively to the inner and outer regions, and 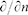 denotes the normal derivative, which only depends upon the radial coordinate in the case of circular objects. Here, 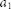 and 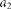 are the inner and outer radii of the shell. Moreover, 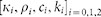 represent the conductivity, fluid density, heat capacity, and wave number in the background medium (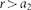), object (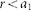), and shell (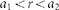), respectively.

### Scattering cancellation technique for heat diffusion waves: static regime

The aim of this study is to show that scattering from various spherical objects can be reduced drastically by carefully choosing the values of the shell conductivity and the specific heat capacity. First, a spherical object centered at the origin of a spherical coordinate system is considered. Two parallel plates set at different temperatures 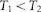, generate a heat flux (plane heat diffusion wave) that impinges on the scattering object [Fig. 1(a)]. In this first section, the case of static (steady-state) regime is considered, i.e. 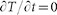. So Eq. (1) is simplified to 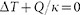. The scalar temperature field 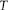 in the different regions of space can be expressed in spherical coordinates (Fig. 1) as,
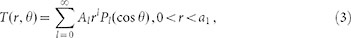

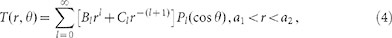

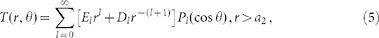
where 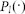 represents the Legendre polynomial of order 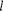. For 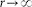, 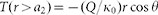, therefore 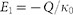 and all the other coefficients 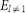 are zero. The remaining coefficients are obtained by solving the linear system
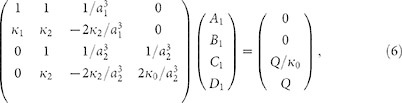
which is obtained by applying the continuity conditions at the boundaries 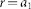 and 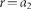. The scattering cancellation condition is obtained by enforcing that the first scattering coefficient 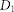 is zero,
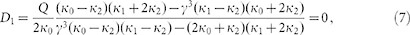
where 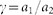. Solving Eq. (7) for 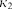 yields the value of the shell conductivity, which ensures that there is no temperature perturbation with a uniform temperature gradient, as if the object does not exist,
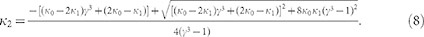


### Scattering cancellation technique for heat diffusion waves: time-harmonic regime

The scattering coefficients relate the scattered fields to the incident ones, and depend on the geometry of the object and the frequency 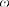. Moreover, for a given size 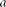 of the object, only contributions up to a given order 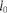 are relevant, since the amplitude of the scattering coefficients changes as 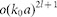. The incident heat excitation is an oblique plane diffusion wave, of incidence angle 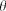, and is of the form 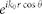. In a spherical coordinate system, it can be expressed as
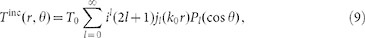
where 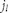 denotes the 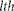 spherical Bessel function and 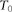 is the amplitude of the incident temperature field. The scattered field (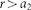) can be expressed in a spherical coordinate system as
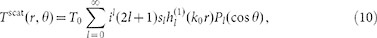
where 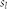 are the complex scattering coefficients and 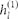 are spherical Hankel functions of the first kind. Therefore, the temperature field can be expressed in the different regions of space as
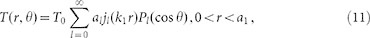

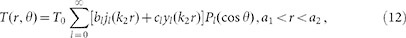

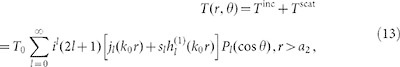
with 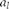 and (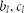) complex coefficients of the temperature field inside the object and the shell, respectively. Applying the continuity conditions at the boundaries 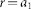 and 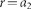 yields the different coefficients. In particular, 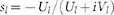. Here 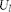 and 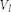 are given by the determinants
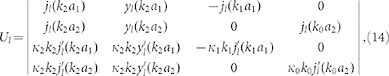
and
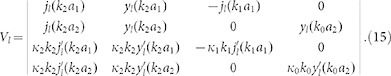
The scattering cross-section (SCS) 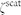 is a measure of the overall visibility of the object to external observers. It is obtained by integrating the scattering amplitude 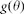, defined such that 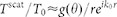, for 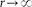,
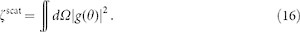
Here, 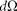 is the incremental solid angle, in spherical coordinates, 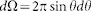, and 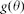 is expressed as
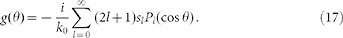
Inserting Eq. (17) into Eq. (16) yields
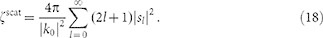
In the quasistatic limit (long diffusion length 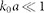), only few scattering orders contribute to the overall scattering cross-section, namely the first two orders (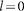 for the monopole, and 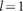 for the dipole mode, unlike in the electrodynamic case, where the first dominant mode is the dipole one). In this scenario, one has
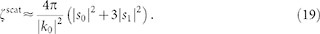
Consequently, canceling these two modes, i.e. 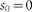 and 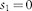, will ensure that 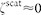, and the thermal scattering from the object will be suppressed. Namely, the SCT conditions on the parameters of the cloaking shell 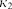, 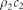, and 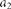 are
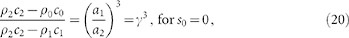
and
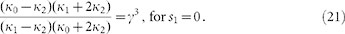
The monopole SCT condition in Eq. (20), depends only on the product of the density and the specific heat of the shell, and the ratio of radii of the object and the shell 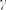. Similarly, the condition in Eq. (21) depends only on the conductivity of the shell and 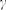. By enforcing these two conditions, the total scattering from the spherical object can be suppressed in the quasistatic limit.

**Figure 2:**
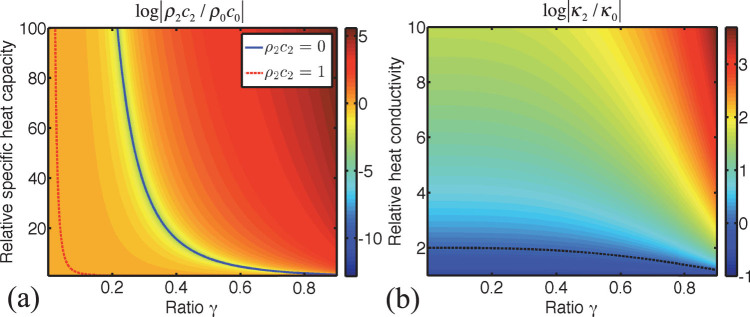
Optimal cloaking parameters. (a) Relative specific heat capacity of the shell 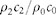 in logarithmic scale, versus the ratio 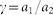 and the relative specific heat capacity of the object 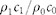. The color bar denotes the plot of 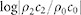. (b) Relative heat conductivity of the shell 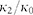 in logarithmic scale, versus the ratio 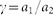 and the relative heat conductivity of the object 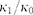. The color bar denotes the plot of log 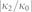
and the dashed black line represents log 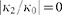.

Figures 2(a) and 2(b) illustrate numerical solutions to Eqs. (20) and (21), where the variation of the relative specific heat capacity 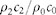 and the relative heat conductivity 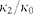 are plotted versus 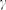 and 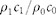 and 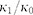, respectively. From the solution of Eq. (20), given in Fig. 2(a), one can see that the relative specific heat capacity of the shell 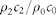, given here in logarithmic scale, takes positive and negative values, depending on 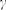 and the heat capacity of the object. The red line represents the curve obeying the equation 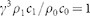 implying 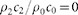. The specific heat capacity takes negative (positive near-zero) values above (below) this curve. From the solution of Eq. (21), given in Fig. 2(b), it can be seen that the required relative heat conductivity of the shell 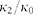 needs to be almost always negative, for varying 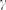 and 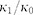. However, for an object with small heat conductivity and small radius [lower part of Fig. 2(b), in blue color], the required shell conductivity is close to zero. In fact, from Eq. (21), one can derive that for the negative solution of Eq. (21)
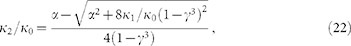
where 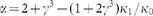. It can be clearly seen that for positive conductivities of the object, the condition 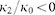 has to be satisfied to achieve the optimal heat cloaking effect.

**Figure 3:**
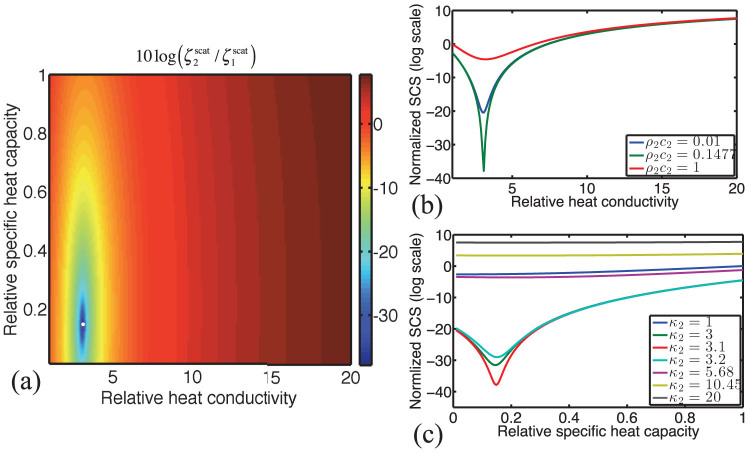
Thermal scattering reduction. (a) Normalized (analytical) SCS 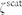 in logarithmic scale, versus the relative heat conductivity 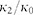 and the relative specific heat capacity 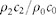. The white dot represents the position of optimized scattering reduction, with a value of 40 dB. The color bar denotes the plot of 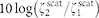, where the subscripts 1 and 2 refer to the scattering cross-section of the obstacle and cloaked structure, respectively. (b) Normalized SCS versus the relative heat conductivity for various values of the specific heat capacity 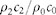. (c) Normalized SCS versus the relative specific heat capacity 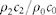 for various values of the relative heat conductivity 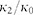.

Let us move now to the analysis of a specific scenario, where the heat scattering of a spherical object is characterized. The relative specific heat capacity of the object is 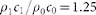 and its relative conductivity is 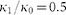. The radius of the object 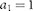, and the wave numbers are normalized to 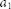. The free space wave number is chosen as 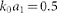. This object is coated with a shell of outer radius 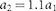. 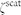 of the total object-shell structure, defined in Eqs. (16)–(18), is normalized to the SCS of the bare object, and plotted against varying values of 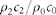 and 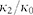. The result is shown in Fig. 3(a) in logarithmic scale. The blue regions correspond to significant scattering reduction, whereas red regions correspond to enhanced scattering from the structure. It can be noticed that ranges of 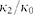 between 1 and 4, and 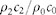 between 0.05 and 0.5, are best for thermal scattering cancellation (now using the positive solution of Eq. (21), for practical realizations). The white dot has coordinates (3.1, 0.15) that correspond to the theoretical SCT condition obtained from Eqs. (20) and (21). It is also interesting to note that numerical simulations taking into account many scattering orders, give scattering reduction of 40 dB, sensibly around the same point.

These results show the importance of taking into account both the shell conductivity and specific heat capacity, in contrast to previous studies that only considered the effect of conductivity through the static analysis. This can be better understood from Figs. 3(b) and 3(c), where the normalized SCS is plotted versus 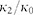 for various values of 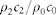, and versus 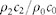 for various values of 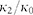, respectively. The sensitivity to variations in 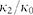 is more evident from these figures, since a small variation from the optimum value results in fast deterioration of the scattering reduction: when 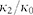 is equal to 1 or 5, there is no dip in the SCS and the scattering is high, as can be seen from Fig. 3(c). The sensitivity to variations in 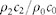 is less important, as can be seen from Fig. 3(b), but it is important to choose values around those predicted by Eqs. (20) and (21). On the other hand, when 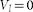, peaks corresponding to modal resonances start appearing in the scattering cross-section (related to Fano-like response of the system due to interference between dark and bright scattering modes)^47^.

**Figure 4:**
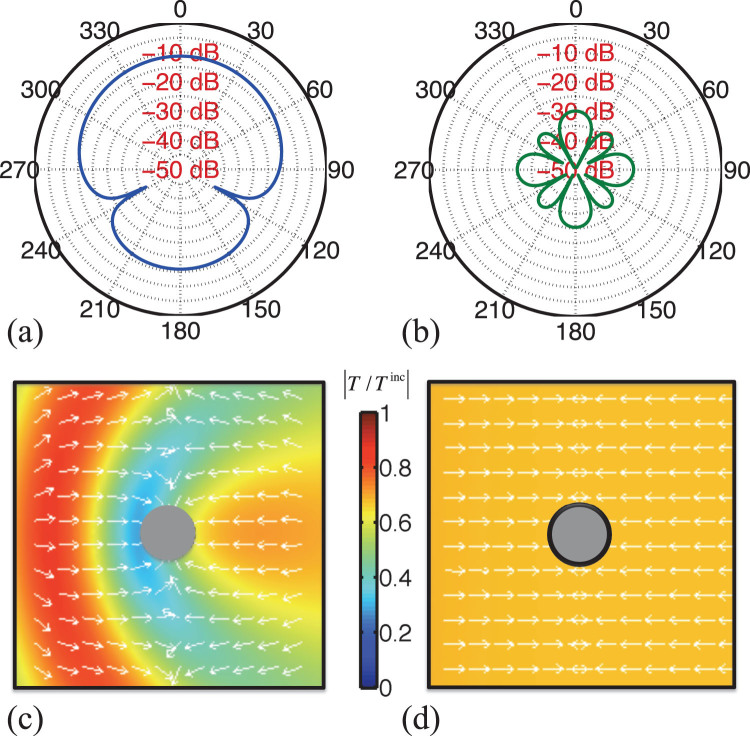
Near and far-field characterization. Analytical scattering amplitude 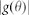, given by Eq. (17), in polar coordinates, and in logarithmic scale (a) for the bare object with 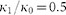 and (b) for the cloaked object, with 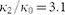. Amplitude of the oscillating temperature in the near-field of (c) the same bare object of Fig. 4(a) and (d) the same cloaked object of Fig. 4(b) for 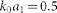. Arrows show the direction of 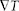 and the color bar denotes the plot of 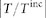.

To better illustrate the efficiency of the proposed cloak, the far-field scattering patterns, i.e. the heat scattering amplitude 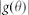 in polar coordinates, in the 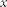
−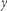 plane, are shown in Figs. 4(a) and 4(b). These figures demonstrate that the object is almost undetectable at all angles with scattering amplitude orders of magnitude lower than that of the bare object. As a result, there is no temperature perturbation around the object immersed in the thermal fields. To further demonstrate the functionality of the cloak, Figs. 4(c) and 4(d) plot the amplitude distribution of the scattered thermal field when the heat from the infinite sheet of oscillating heat source is impinging from left to right on the structure, without and with the cloaking shell, respectively. When the object is cloaked, the field amplitude is constant everywhere in space in contrast to the case of the object without the cloak.

## Discussion

### Mantle cloaking for heat diffusion waves

**Figure 5:**
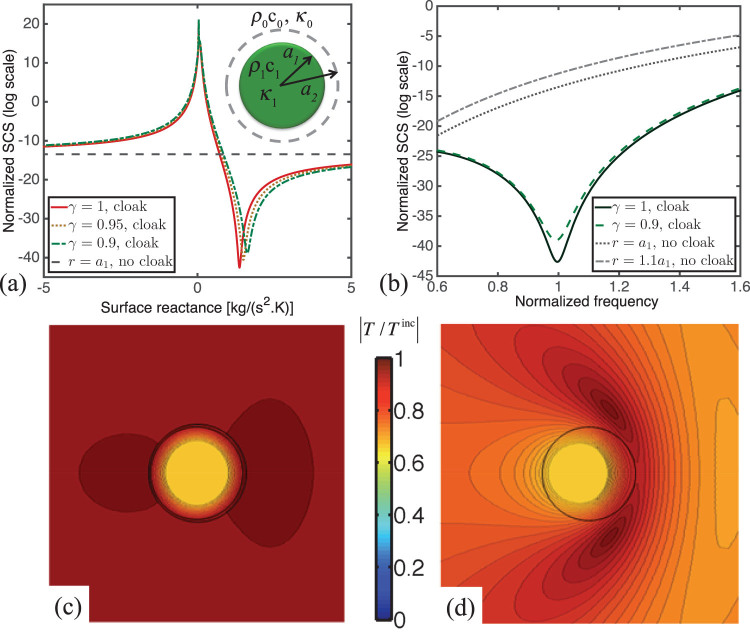
Thermal mantle cloaking. Normalized (analytical) SCS 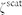 versus 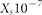 for the object with normalized radius 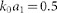 and relative heat conductivity 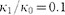 for various values of the ratio 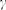. (b) Normalized SCS 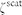 versus normalized frequency for the same object with 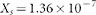 and 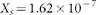 for values of the ratio 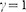 and 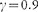. The inset of Fig. 5(a) illustrates the object coated with a thermal metasurface, projected in the 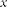
−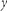 plane. Amplitude of the temperature field on the 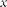
−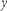 plane for the same object (c) with mantle cloak with 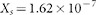 and (d) without the cloak. The color bar denotes the plot of 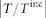.

As stated in the introduction, recent findings suggest that objects can be made invisible using the surface cloaking technology, where a metasurface may produce similar cloaking effects in a simpler and thinner geometry^14^,^15^,^16^. The ultrathin mantle cloak with an averaged surface reactance metasurface^13^ reduces the scattering from the hidden object, comparable to bulk metamaterial cloaks. The setup of the problem is similar to the previous section, except for the fact that scattering cancellation is achieved by a surface, instead of a shell. This is illustrated in the inset of Fig. 5(a). The impedance boundary condition results in jumps in the radial component of the density of heat flux, on the interface between the two media.

In what follows it is shown that the scattering from various spherical objects can be drastically reduced by choosing the appropriate surface impedance, and thus their visibility to heat diffusion waves can be suppressed.

To design a mantle cloak, we keep the boundary conditions at 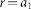 same as those in the previous sections, while we replace the boundary conditions at 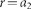 with
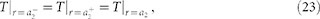

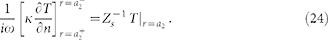
Eq. (24) is a surface impedance condition that implies a jump in the density of heat flux. Here, 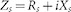 is the averaged surface impedance that relates the temperature to the density of heat flux on the surface.

Following the procedure described in the previous section, with these new boundary conditions, one can show that the 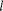
th spherical scattering harmonic can be suppressed, provided that the following determinant is canceled,
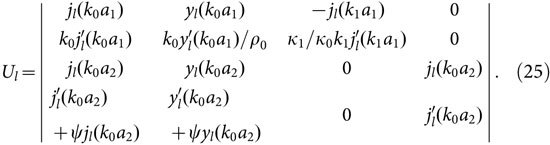
It should be noted that for the mantle cloak design considered here, 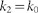 and 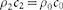. In Eq. (25), the dimensionless function 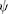 is defined as
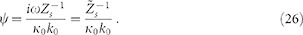
For 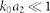, the spherical Bessel functions take a simpler polynomial form, and the approximate cloaking condition in this limit can be written as
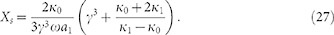
This clearly shows that by properly choosing the thermal surface reactance (expressed in units of J = (m^2^K)), it is possible to suppress the dominant multipolar scattering in the quasistatic limit.

Figure 5(a) plots the SCS versus 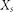 for cloaked objects with various 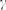. The SCS of a bare object is plotted for comparison. For 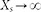, we notice that the metasurface does not reduce the scattering, consistent with the limit of no-surface. For specific values of 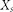, however, a relevant scattering reduction is achieved, and this may be obtained for different values of 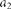, even in the limit of a cloak winding conformal to the object (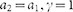).

Figure 5(b) plots the SCS versus the frequency for cloaked objects with 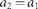 (conformal) and 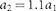. We suppose here that the surface reactance does not vary with frequency and is given with 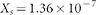 for 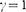 and 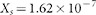 for 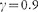. The SCS of uncloaked objects with radius 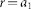 and 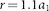 are plotted for comparison. It is evident that excellent scattering reduction may be achieved over a large range of frequencies for both cases.

Figures 5(c) and 5(d) plot the amplitude of the temperature field scattered by a cloaked and uncloaked object, on the 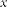
−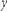 plane at a time instant, respectively. When the object is cloaked, both forward and backward scattering almost vanish. This reduction of scattering is achieved due to the proper choice of the surface impedance, which restores almost uniform amplitude all around the cloak.

### Summary

In conclusion, we have proposed an original route towards designing thermal cloaks based on the scattering cancellation technique. This technique is inspired by the plasmonic cloaking, which makes use of shells with induced negative polarization to suppress scattered electromagnetic fields. And contrary to invisibility cloaks based on transformation optics, SCT offers simple cloaking designs (without the need of anisotropy and inhomogeneity of the physical parameters).

One may envision that using this design may further make the thermal cloaking closer to its practical and feasible realization. We believe that such a structured cloak could be manufactured within current technology, having in mind some potential applications in invisibility, sensing and thermography. The range of industrial applications is vast, and our proof of concept should foster research efforts in this emerging area of thermal cloaks and metamaterials.

## Methods

Analytical methods based on scattering Mie theory of spherical thermal scatterers are used to obtain the results presented in Figs. 2, 3, 4(a), 4(b), and 5. In the quasistatic limit, where the size of the object is much smaller than the wavelength and only the lowest-order Mie coefficients are kept, analytical formulas are obtained [Eqs. (20) and(21)]. Those give results similar to the ones obtained from full Mie series solutions [Fig. 3(a)]. The results given in Figs. 4(c) and 4(d) are obtained using COMSOL Multiphysics software, which solves Eq. (2) with proper boundary conditions using a finite element scheme.

## Author Contributions

M.F. and P.-Y.C conceived the idea of this study. M.F. performed numerical simulations and wrote the manuscript. P.Y.C., H.B., C.A., S.G., and A.A. contributed to the analysis of the results and reviewed the manuscript. S.G. and A.A. supervised the project.
