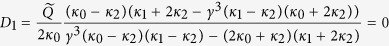# Corrigendum: Thermal invisibility based on scattering cancellation and mantle cloaking

**DOI:** 10.1038/srep19321

**Published:** 2016-01-21

**Authors:** M. Farhat, P.-Y. Chen, H. Bagci, C. Amra, S. Guenneau, A. Alù

Scientific Reports 5: Article number: 987610.1038/srep09876; published online: 04302015; updated: 01212016

This Article contains errors.

In the Results section under subheading ‘Scattering cancellation technique for heat diffusion waves: static regime’

“For 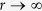, 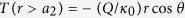, therefore 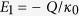 and all the other coefficients 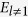 are zero.”

should read:

“For 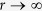, 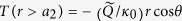 with 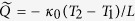 the heat generated by unit surface and unit time, in contrast to *Q*, of Eqs (1)–(2) that represents the heat generated by unit volume and unit time. Therefore 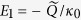 and all the other coefficients 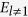 are zero.”

In Equation (6),


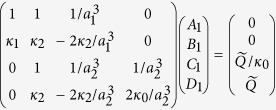


should read:


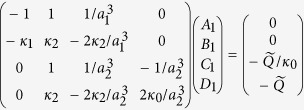


And lastly, in Equation (7)


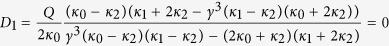


should read: